# Supplementary material for: Before Direct-Acting Antivirals for Hepatitis C Virus: Evaluation of Core Protein R70Q and L/C91M Substitutions in Chronically Infected Brazilian Patients Unresponsive to IFN and/or RBV
Source: Viruses. 2023 Jan 9;15(1):187. doi: 10.3390/v15010187 (PMC9863677; doi:10.3390/v15010187)
Supplement: Supplementary file 1 [file viruses-15-00187-s001.zip › viruses-2116279-supplementary.docx]

Article

Before Direct-Acting Antivirals for Hepatitis C Virus:
Evaluation of Core Protein R70Q and L/C91M Substitutions
in Chronically Infected Brazilian Patients Unresponsive to
IFN and/or RBV

Letícia Bomfim Campos ^1^, Nathália Alves Araújo de Almeida ¹, Catarina Góis de Santana ^2^,
Evorah Nascimento Pereira Barbosa ^1^, Marco Aurelio Pereira Horta ^3^, Márcia Amendola Pires ^2^, Carlos Eduardo Brandão Mello ², Vanessa Salete de Paula ¹ and José Júnior França de Barros ^1,^*

**Table S1.** Pyrosequencing result of the substitution L91M.

| **Sample** | **Subgenotype** | **Position** | **Sanger**  **Sequencing*** | **Pyrosequencing** | |
| --- | --- | --- | --- | --- | --- |
|  |  |  |  | **%T/CTG (Leucine)** | **%ATG**  **(Methionine)** |
| 5 | 1b | 91 | M (M) | 0 | 100 |
| 7 | 1b | 91 | M (M) | 0 | 100 |
| 11 | 1b | 91 | M (M) | 0 | 100 |
| 22 | 1b | 91 | M (M) | 0 | 100 |
| 27 | 1b | 91 | M (M) | 0 | 100 |
| 34 | 1b | 91 | M (M) | 0 | 100 |
| 37 | 1b | 91 | M (M) | 0 | 100 |
| 38 | 1b | 91 | M (M) | 0 | 100 |
| 39 | 1b | 91 | M (M) | 7 | 93 |
| 41 | 1b | 91 | M (M) | 5.5 | 94.5 |
| 44 | 1b | 91 | M (M) | 0 | 100 |
| 53 | 1b | 91 | **L (W)** | 100 | 0 |
| 55 | 1b | 91 | M (M) | 0 | 100 |
| 65 | 1b | 91 | M (M) | 0 | 100 |
| 66 | 1b | 91 | M (M) | 0 | 100 |
| 67 | 1b | 91 | M (M) | 0 | 100 |
| 71 | 1b | 91 | M (M) | 0 | 100 |
| 74 | 1b | 91 | L (W) | 100 | 0 |
| 75 | 1b | 91 | M (M) | 0 | 100 |
| 76 | 1b | 91 | M (M) | 0 | 100 |
| 78 | 1b | 91 | M (M) | 0 | 100 |
| 84 | 1b | 91 | M (M) | 0 | 100 |
| 86 | 1b | 91 | M (M) | 0 | 100 |
| 87 | 1b | 91 | M (M) | 0 | 100 |
| 91 | 1b | 91 | M (M) | 0 | 100 |
| 92 | 1b | 91 | M (M) | 0 | 100 |
| 94 | 1b | 91 | **L (W)** | 58 | 42 |
| 98 | 1b | 91 | M (M) | 0 | 100 |
| 104 | 1b | 91 | M (M) | 0 | 100 |
| 105 | 1b | 91 | M (M) | 1 | 99 |
| 107 | 1b | 91 | M (M) | 5.5 | 94.5 |
| 111 | 1b | 91 | M (M) | 0 | 100 |
| 116 | 1b | 91 | M (M) | 2.5 | 97.5 |
| 120 | 1b | 91 | M (M) | 0 | 100 |
| 121 | 1b | 91 | M (M) | 0 | 100 |
| 136 | 1b | 91 | M (M) | 2.5 | 97.5 |
| 137 | 1b | 91 | M (M) | 2.5 | 97.5 |
| 138 | 1b | 91 | M (M) | 3.5 | 96.5 |
| 145 | 1b | 91 | M (M) | 1.5 | 98.5 |
| 147 | 1b | 91 | M (M) | 1 | 99 |
| 151 | 1b | 91 | M (M) | 0 | 100 |
| 159 | 1b | 91 | **L (W)** | 100 | 0 |
| 175 | 1b | 91 | M (M) | 0 | 100 |
| 183 | 1b | 91 | M (M) | 0.5 | 99.5 |
| 187 | 1b | 91 | M (M) | 0 | 100 |
| 207 | 1b | 91 | M (M) | 0 | 100 |
| 208 | 1b | 91 | **L (W)** | 96 | 4 |
| 210 | 1b | 91 | M (M) | 1 | 99 |
| 211 | 1b | 91 | M (M) | 0 | 100 |
| 212 | 1b | 91 | M (M) | 0 | 100 |
| 233 | 1b | 91 | **L (W)** | 2.5 | 97.5 |
| 234 | 1b | 91 | M (M) | 4.5 | 95.5 |
| 235 | 1b | 91 | **L (W)** | 100 | 0 |
| 241 | 1b | 91 | M (M) | 4.5 | 95.5 |
| 248 | 1b | 91 | M (M) | 0 | 100 |
| 249 | 1b | 91 | **L (W)** | 3.5 | 96.5 |
| 268 | 1b | 91 | **L (W)** | 96 | 4 |
| 269 | 1b | 91 | M (M) | 4.5 | 95.5 |
| 280 | 1b | 91 | **L (W)** | 100 | 0 |
| 282 | 1b | 91 | **L (W)** | 100 | 0 |
| 285 | 1b | 91 | M (M) | 0 | 100 |
| 289 | 1b | 91 | **L (W)** | 1.5 | 98.5 |
| 293 | 1b | 91 | **L (W)** | 100 | 0 |
| 294 | 1b | 91 | M (M) | 0.5 | 99.5 |
| 295 | 1b | 91 | M (M) | 8.5 | 91.5 |
| 297 | 1b | 91 | M (M) | 0 | 100 |
| 298 | 1b | 91 | M (M) | 1 | 99 |
| 302 | 1b | 91 | M (M) | 12 | 88 |
| 306 | 1b | 91 | **L (W)** | 100 | 0 |
| 310 | 1b | 91 | **L (W)** | 100 | 0 |
| 318 | 1b | 91 | M (M) | 0 | 100 |
| 323 | 1b | 91 | **L (W)** | 97.5 | 2.5 |
| 331 | 1b | 91 | M (M) | 3.5 | 96.5 |

*The classification of mutant or wild samples is based on Sanger sequencing results in grey for isolate 94.
